# Analysis of the Algae Growth Dynamics in the Hydroponic System with LEDs Nighttime Lighting Using the Laser Granulometry Method

**DOI:** 10.1007/s11270-018-4075-8

**Published:** 2019-01-07

**Authors:** A. Bawiec, T. Garbowski, K. Pawęska, K. Pulikowski

**Affiliations:** Institute of Environmental Engineering, Wrocław University of Environmental and Life Sciences, 24 Grunwaldzki Sq., 50-363 Wrocław, Poland

**Keywords:** Laser granulometer, Algal biomass, Fractal dimension, Hydroponic wastewater treatment, Wastewater treatment

## Abstract

The latest research focused on the analysis of algal growth and the dynamics of their growth use the laser diffraction technique, enabling determination of the volume fraction of suspended particles with specific diameters in aqueous solution as well as their fractal dimensions. This study focuses on the possibility of using a laser granulometer to assess the growth dynamics of algae growing in treated wastewater in a hydroponic system, supported by artificial lighting with the use of light-emitting diodes (LEDs). On the basis of the measurements, the fractal dimension (Df) of algae was determined. An attempt was made to apply the modified Avrami equation describing the crystallization process for the analysis of algae growth dynamics in wastewater. Presented results show that the fractal dimension of suspended matter, largely created by algae, in the case of additional lighting of the hydroponic system at night, takes lower values (Df ~ 1.0) than in sewage without additional light source (Df ~ 2.0). In each measurement series, the fractal dimension of particles in the tank with lighting in the end of the experiment was about 33–43% lower than in the tank without LEDs. The analysis of changes in particle diameters calculated on the basis of Avrami equation largely corresponds with the stages of algae growth. During the measurement series with lower air temperatures, the growth of algae in the tank with additional light was faster than in the tank without LEDs. The obtained information can be the basis for determining the effective method of removing algae from wastewater treated in the hydroponic system, before they are discharged to the receiver in order to prevent the outflow of increased concentrations of total suspended solids.

## Introduction

Eutrophication as the process of water enrichment with nitrogen and phosphorus loads is currently one of the most serious environmental problems and subject of study of scientists around the world (Andersen et al. 2017). This problem applies not only to freshwater reservoirs, but also to watercourses, groundwater, seas, and oceans (Andersen et al. 2017; Kelly et al. 2018; Kubicz et al. 2018). The most important role in the development of the eutrophication process have nutrients (nitrogen and phosphorus compounds) flowing into the water (Dupas et al. 2015). One of the reasons for the increased inflow of N and P to rivers and other watercourses is the discharge of insufficiently treated wastewater into the receiving water bodies (Dąbrowska et al. 2017). This problem may increase due to continuous population growth as well as increased urbanization and industry development, and thus, an increased amount of municipal and industrial wastewater (Szewrański et al. 2018). The consequence of the occurrence of eutrophication is ecosystem damage due to the massive development of algae (including cyanobacteria), low dissolved oxygen concentrations and undesirable pH changes (Christenson and Sims 2011).

An increasingly popular method of wastewater treatment aimed at more effective reduction of nutrients at the outflow from the sewage treatment plant is the hydroponic method. The treatment of wastewater with its use consists in redirecting biologically treated wastewater (activated sludge, biological membrane) to the hydroponic ditch, built in the shape of an artificial river. On the surface of sewage flowing through the ditch (hydroponic lagoon), water plants are planted—macrophytes to uptake nitrogen and phosphorus from the flowing solution (Kouamé et al. 2016). Of great importance for processes of purification in such systems are bacteria that colonize floating panels and plant root system, small invertebrates, and above all, algae (Ye et al. 2016). Hydroponic systems used as tertiary treatment significantly support the removal of organic compounds from wastewater and are a much cheaper alternative to chemical methods of sewage treatment.

Algae growing in wastewater treated in a three-stage system significantly support the processes of their purification due to the possibility of using inorganic forms of nitrogen and phosphorus in the processes of biomass growth. They also have the ability to uptake heavy metal ions, organic pollutants, and coliform bacteria, thus protecting the receiver of treated wastewater from pollution (Abdel-Raouf et al. 2012). The great advantage of algae is their very fast growth rate and short generation times, thanks to which they are able to double their mass within 24 h, and thus, take nutrients from the solution (Demirbas 2011; Dogaris et al. 2015).

The use of algae in wastewater treatment is an eco-friendly solution, provided that their growing biomass is removed from the system and reused (Munoz and Guieysse 2008). Wastewater is often an ideal nutrient solution for the development of specific species of algae used for the production of biofuels (Rawat et al. 2013).

The most difficult task associated with the use of algae for the wastewater treatment or their cultivation for energy purposes is their removal from the system. Algae, due to their small size, mobility, negatively charged surfaces, storage of lipids in cells, and their low density, in aqueous solution, remain generally suspended (Sathe and Durand 2015; Garbowski et al. 2017a). Common methods of algal biomass harvesting are centrifugation, filtration, chemical flocculation, and sedimentation. However, these are costly methods, based on the use of chemical substances, which discharged into the environment without proper treatment and disposal, pose a serious threat to the ecosystem (Milledge and Heaven 2013). One of the biological methods of removing algae from wastewater can be the use of zooplankton, which is an effective method and does not require the use of chemical substrates (Sathe and Durand 2015).

Each method of removing algae from an aqueous solution requires obtaining information on the size, shapes, and other properties of the particles being removed. For sedimentation and filtration processes, it is required to obtain microalgae aggregates or stable algae-bacterial flocs, which size exceeds 50 μm. The occurrence of small, single algae cells (1–10 μm) make the sedimentation process impossible. Large diameters of algae (50–200 μm) prevent their uptake by most species of zooplankton (Montemezzani et al. 2015). Therefore, in order to choose the method of removing algae from wastewater treated in the hydroponic system before they are discharged to the receiver, there is a need to monitor processes of algae growth.

A novel method of assessing the dynamic growth of algae in biologically treated wastewater is the use of a laser granulometer (Garbowski et al. 2017b; Bawiec et al. 2017). Laser granulometry is used for analysis of particle size distribution in aqueous solutions. This method is based on the phenomenon of diffraction and low angle light scattering (Tinke et al. 2008). The beam of red and blue light emitted by the laser flows through the measuring cell where the sample is located. After that, light beam is dispersed on the particles contained in the sample. Detectors measure the intensity of scattered light and allow the identification of particle size (Sperazza et al. 2004; Kuśnierz and Łomotowski 2015).

The fractal dimension obtained as a result of numerical transformations of data gained during the measurements informs about the shape of the particles contained in the solution. Particles of suspended solids dispersed in the medium such as sewage tend to aggregate—they combine into larger particles. Determination of the fractal dimension of particles (Df) makes it possible to assess the density and porosity, as well as the degree of particles thickness. It is possible to assess whether aggregates are strongly related to each other creating structures similar in shape to the sphere or create a compact cluster (Zhao et al. 2013; Kuśnierz and Wiercik, 2016). In the study of wastewater treated in the hydroponic system, the particles of suspended solids investigated using a laser granulometer have predominantly diameters corresponding to the diameters of various species of algae present both in the form of single cells and colonies (aggregates). The basis for determining the fractal dimension using laser technology is the assumption that the angle of light scattering on suspension particles depends on the distribution of their size, optical properties and spatial structure. It is particularly effective for particles with low refractive index values and loose structure (Wilén et al. 2003; Bushell 2005).

It is assumed that to initiate the process of algae colonies development, it is often enough to have single cells in the sewage and to ensure appropriate conditions (access of light, appropriate temperature, and pH). To identify and describe the algae growth process in the hydroponic system, the information about the course of the crystallization process described by the Avrami equation might be used (Kuśnierz and Łomotowski 2015). The crystallization theory assumes that the crystal structure is created by adhesion of single cells to the crystallization nuclei, what can be also observed in the growth of algae aggregates.

The aim of this study was to evaluate the dynamics of algae growth in the wastewater treated with hydroponic method, to determine the effect of LED lighting on the development of algae biomass. The algae growth process observed with the use of laser granulometer may give the relatively fast information about the size of algae cells or aggregates. The basic knowledge about algae size allows to choose a method for algae removal from the hydroponic system to avoid excess contamination of receiving water body by suspended solids.

## Materials and Methods

### Experimental Set-up

**Fig. 1:**
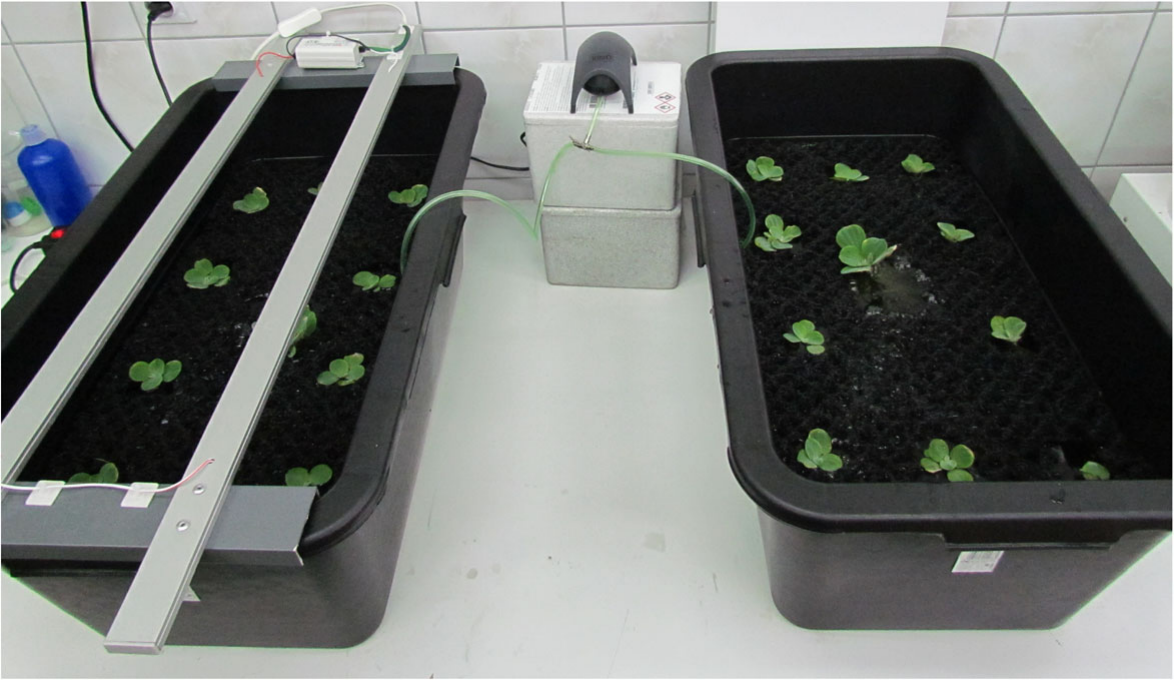
Experimental set-up—tank with additional light source (on the left) and without artificial lighting [phot. A. Bawiec]

Experimental set-up consisting of two polyethylene tanks with opaque walls was located in the laboratory at the Institute of Environmental Engineering of the Wrocław University of Environmental and Life Sciences. The 60-dm^3^ tanks were filled up with 30 dm^3^ of wastewater treated in the biological reactor. Concentration of total nitrogen during the experiment ranged from 12.05 to 16.93 mgN dm^−3^ while the phosphorus concentration was 1.2–3.7 mgP dm^−3^. To avoid anaerobic decomposition of organic matter, the sewage was constantly aerated. Dissolved oxygen (DO) rate was maintained on the level of 9.5–10.8 mgO_2_ dm^−3^. DO measurements were made with the use of portable oxygen meter (oxygen probe, HI9146-04). On the surface of the wastewater, *Pistia stratiotes* seedlings were planted to create conditions close to the hydroponic lagoon working in municipal wastewater treatment plant as a third stage of treatment. In each tank, the number of seedlings was the same (11 plants) with a similar number of leaves. One of the tanks was equipped with artificial light source, aluminum rail with light-emitting diodes (LEDs) in the color of red (625–675 nm) and blue (425–475 nm) that corresponds to the main absorption peaks of chlorophyll a and b (Gupta 2017). The ratio of the red light to the blue light was like 4:2. The lighting time was set from the sunset to sunrise with the use of time switch. Air temperature during the first measurement series oscillated within 15–20 °C, and during the second measurement series within 16.5–22 °C. Experimental set-up is presented in Fig. 1.

### Sampling and Granulometric Analyses

During the experiment, two series of measurement (two repetitions) lasting 56 days were made. The pH of wastewater in the tanks was in range of 8.50–8.73. Samples for granulometric analyses were collected at the beginning of experiment and then every 2 weeks. Every time, 800 ml of sample was taken from each of the tanks after thorough mixing of wastewater. The measurements were made with the use of the Malvern Mastersizer 2000 laser granulometer correlated with the computer and Malvern Instruments Ltd. software. The Malvern device is equipped with the wet sample dispersion unit “Hydro MU” that allows to obtain homogeneous sample of tested solution or mixture. Homogenization of the sewage sample was obtained using a stirrer with 1500 rpm speed. Each sample was characterized by a low suspension concentration and low turbidity so the obscuration did not exceed 10%. After the measurements, wastewater were directed back into the tanks.

### Fractal Dimensions Measurements

The fractal dimension of particles (Df) in wastewater taken from the tanks of the experimental set-up were determined based on the analysis of changes in the intensity of the scattering of the laser light wave. On this basis, the relationship between the intensity of light scattering and the wave number was determined. The calculations were performed using an Excel spreadsheet prepared by Malvern Instruments Ltd., with a restricted computational procedure to determine the angle of the straight line in a double logarithmic system according to the dependence Eq.(1):

1 $$ \left(\mathrm{logI}\left(\mathrm{Q}\right)-\mathrm{logQ}\right) $$

where:

I(Q): Intensity the laser light wave scattering
Q: Wave number

### Avrami Equation and Formation of Algae Aggregates

The modified Avrami equation can be used to describe the algae agglomeration process, as demonstrated by Kuśnierz and Łomotowski (2015) and confirmed by Garbowski et al. (2017b). The modified Avrami equation is described in the following equation Eq. (2):

2 $$ \mathrm{V}\left(\mathrm{d}\right)=1-\exp\ \left(-k\times {\mathrm{d}}^n\right) $$

where *k*’ depends on environmental conditions and exponent *n*’ is associated with the geometry of new products of transformation and can take values from 1 to 4.

The modified form of the Avrami equation uses the dependence of the equivalent diameter of forming crystals on the linear degree of crystal growth over time (Huang et al. 2010).

To determine the empirical regression coefficients described in Eq. (2), a module for non-linear estimation of the Statistica 13.1 program was used.

The calculations were made using the smallest squares function with the Levenberg-Marquardt algorithm, and the non-linear regression accuracy was checked based on the value of the correlation coefficient *R*^2^ (Kuśnierz and Łomotowski 2015).

## Results

Analyses using the modified form of the Avrami equation were made for the values of *k* and *n* (calculated by non-linear estimation) and sizes of diameters ranging from 0.002 to 2000 μm. The analyses were carried out for sewage samples collected periodically during the two measurement series. The volume of particles with a certain diameter in the total volume of the sample was calculated with the use of Eq. (2). The fractal dimensions measurements were made with the use of Eq. (1).

**Fig. 2:**
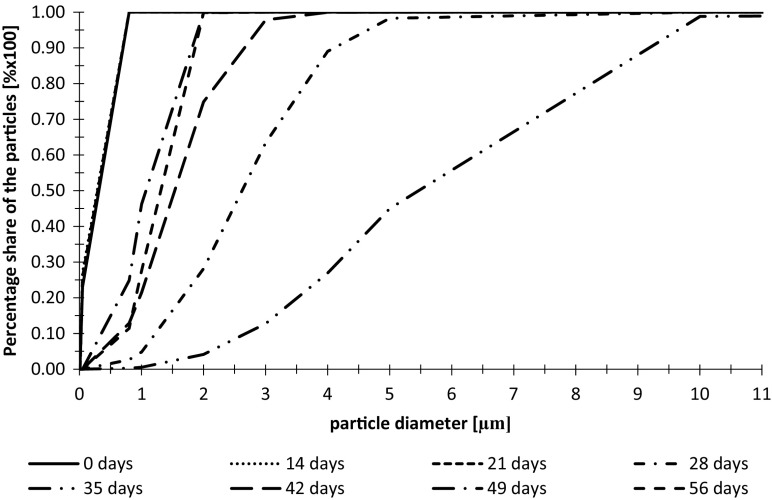
Cumulative volume frequency of particles diameters in wastewater from the tank with additional LED lighting during the first measurement series

**Fig. 3:**
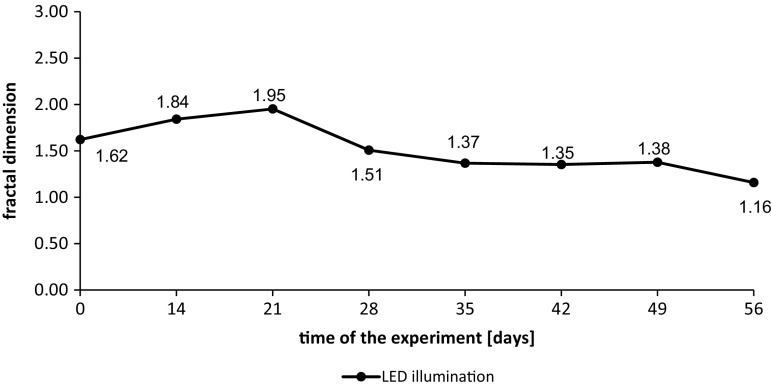
Fractal dimensions of particles in wastewater from the tank with additional LED lighting during the first measurement series

Cumulative volume frequency of particle diameters and fractal dimensions of suspensions in wastewater treated with the use of additional lighting during the first measurement series are shown in Figs. 2 and 3.

As shown in the chart in the first measurement series, the largest particle diameter occurred after 35 days of the experiment. Until day 21, particle diameter was comparatively constant. The increase of diameter was noted after 28 days of experiment. The reason for this might be occurrence of individual cells of algae in the initial phase of the experiment and their aggregation after 28 days.

The fractal dimension of particles in wastewater samples taken from the tank with additional lighting was increasing during the first 21 days of the experiment and reach maximum value Df = 1.95. The increase of Df to the value of almost 2.0 gives an information about expansion of the particles and filling of the entire surface (Łomotowski et al. 2008). After 3 weeks, the fractal dimension was decreasing and in the 56th day reached the lowest value Df = 1.16. The values of coefficient of determination *R*^2^ obtained during calculations of fractal dimensions of particles varied from 0.9825 to 0.9974 with the average value of 0.9940. The values of *R*^2^ indicates a very good match (Schroeder et al. 2016). When the fractal dimension is close to 1.0, the particle shape is linear. Analyses of the fractal dimension changes show that at the beginning of the experiment, the particles of algae were small and concentrated but after 21 days of particle diameter grow, they started to aggregate. The aggregation of algae cells could be the reason of the structure loosening what led to the loss of their spatial structure.

**Fig. 4:**
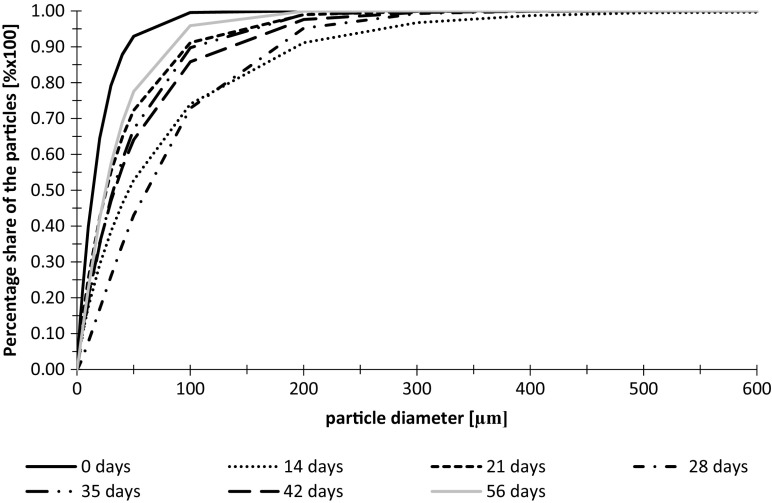
Cumulative volume frequency of particles diameters in wastewater from the tank with additional LED lighting during the second measurement series

**Fig. 5:**
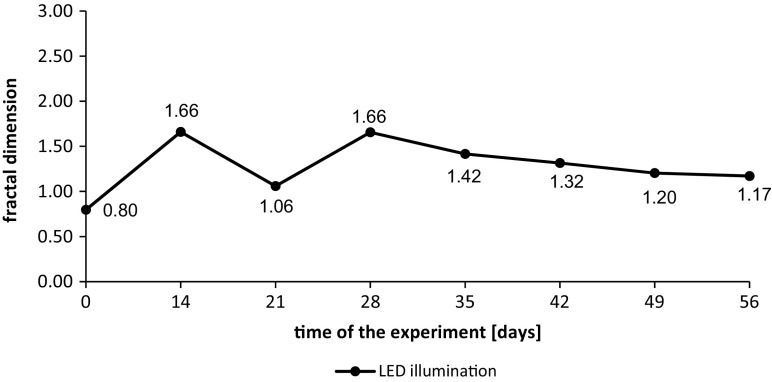
Fractal dimensions of particles in wastewater from the tank with additional LED lighting during the second measurement series

The measurements of particle diameters and the fractal dimensions in wastewater from the tank with additional LED lighting during the second measurement series are shown in Figs. 4 and 5.

In the second cycle of measurements, the rapid growth of algae diameters was observed during first 2 weeks of experiment. In the 21th day, the decrease of particle diameter was observed but during the next two weeks, it started to grow reaching the size of particle diameters similar to day 14. The next drop in particles diameters size occurred in a period from the 35th to 42th day and it was decreasing to the last day.

The fractal dimension changes were similar to the changes of particle diameters. Until the 14th day, when the algae growth was observed, also the degree of algae colony development occurred reaching the Df value of 1.66. In the 21th day of the experiment, the disintegration of algae aggregates occurred. In the next week the fractal dimension grew again same as particle diameter. After 28 days the permanent slow decline of fractal dimension was noted. This phenomenon was connected with slow disintegration of algae aggregates. The values of obtained coefficient of determination in each sample exceeded 0.9800, with the average value of *R*^2^ on the level of 0.9955.

**Fig. 6:**
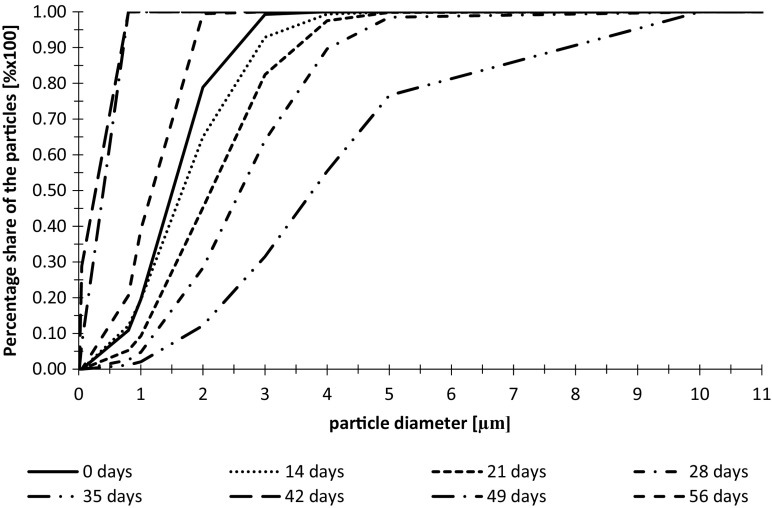
Cumulative volume frequency of particles diameters in wastewater from the tank without additional LED lighting during the first measurement series

**Fig. 7:**
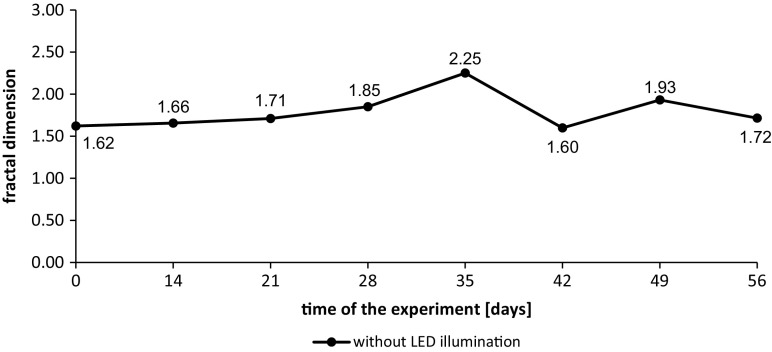
Fractal dimensions of particles in wastewater from the tank without additional LED lighting during the first measurement series

Cumulative volume frequency of particle diameters and their fractal dimensions in wastewater from the tanks without additional LED lighting during the first measurement series are presented in Figs. 6 and 7.

In wastewater from the tank without additional lighting during the first measurement series, very regular gradual increase in particle diameter from 0 to 35 days was observed. It may indicate slow growth of algae aggregates in wastewater. After the 35th day of the experiment, there was a slow decrease in particle diameters what is connected with the breakup of aggregates created in the initial phase of the experiment.

**Fig. 8:**
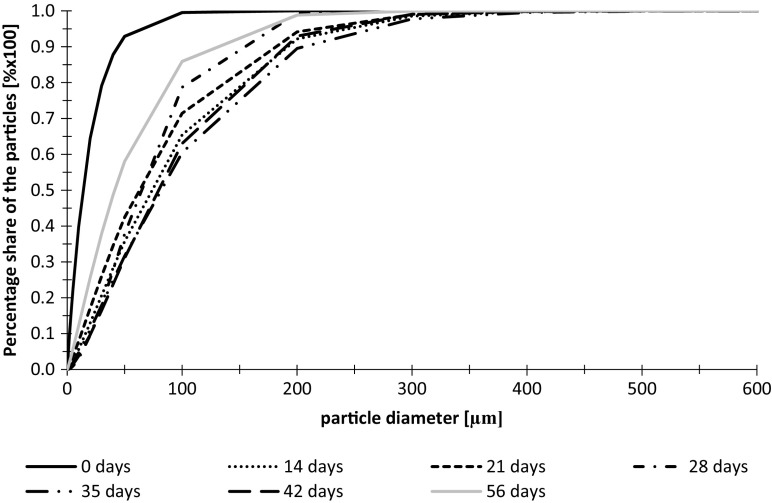
Cumulative volume frequency of particle diameters in wastewater from the tank without additional LED lighting during the second measurement series

Until the 35th day of the experiment, the fractal dimension of particles shows an upward trend as well as their diameters presented in Fig. 6. The values of Df ranged from 1.60 to 2.25. The level of match during Df calculation reached *R*^2^ = 0.9998 (Fig. 8).

**Fig. 9:**
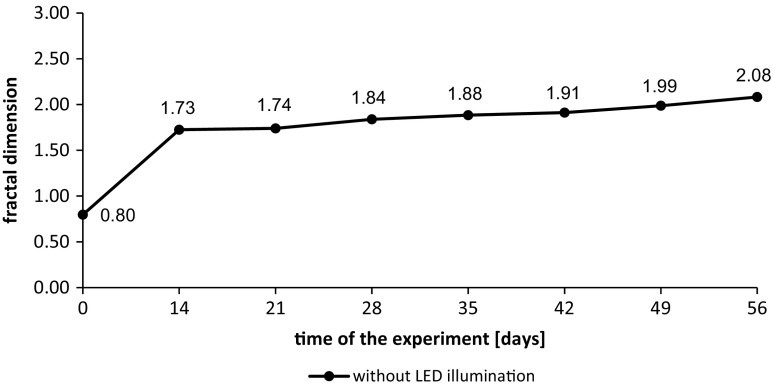
Fractal dimensions of particles in wastewater from the tank without additional LED lighting during the second measurement series

During the second measurement series, higher diameters of suspended solids particles were observed. From the beginning (day 0) to the 14th day of the experiment, rapid growth of the particles diameter was observed that may indicate suitable conditions for the growth of algae aggregates. In the 21st day, small decrease of the diameters was observed but after 35 days, they were growing to reach the highest dimensions. As in the case of wastewater with additional lighting, during the last 2 weeks of experiment, the particles diameter were decreasing that may indicate occurrence of the last phase of algae culture growth (Fig. 9).

Fractal dimension of the particles showed rapid growth after first 2 weeks of the experiment. After the 14th day, the growth of Df proceeded to the end of conducted test. The fractal dimension measured in the sample of wastewater taken from the tank on day 56th was 2.08. Obtained results show that algae aggregates had more spatial structure than at the beginning of the test. The level of match of the intensity of light scattering curve from the light scattering angle which is the basis of fractal dimension calculation reached *R*^2^ = 0.9981.

Based on the information about the distribution of particle diameters the 10th, 50th, and 90th percentile was calculated. The percentile indicates the value below in which 10%, 50%, or 90% of the observations can be found. The data containing measured percentiles (d(0.1), d(0.5), d(0.9)), and fractal dimensions are presented in Table 1.

**Table 1:** Values of particle diameters percentiles and fractal dimension of particles identified in the wastewater during two measurement series

		LED lighting	No LED lighting
	Day 0	Day 56	Day 56
First measurement series			
d(0.1)	0.038	0.860	0.735
d(0.5)	0.072	1.136	1.032
d(0.9)	0.142	1.763	1.847
Df	1.62	1.16	1.72
Second measurement series			
d(0.1)	0.343	0.833	0.710
d(0.5)	0.470	1.092	0.912
d(0.9)	0.888	3.791	2.041
Df	0.80	1.17	2.08

As presented in Table 1 in both of the measurement series after 56 days, the diameters of particles increased in both tanks—with LED and without LED lighting. The amount of particles with higher diameters increased as well. There is no significant difference between percentiles measured for particle diameters in wastewater with and without additional lighting; however, the significant differences can be observed for fractal dimensions. In both cases, the Df vary for lightened wastewater and the sewage from tank without LEDs. The differences are caused by different stages of algae growth in the 56th day of the experiment. In lightened wastewater in both series, few cycles of algae growth occurred when in the sewage without LED in the second measurement series, the growth was constant.

## Discussion

The results obtained during the two series of experiment show differences in dynamics of algae growth in the tanks with and without additional lighting during the night. In the tank without additional light source, the growth of algae colonies was slower than in the reservoirs with LEDs. In the first measurement series, maximum dimensions obtained after 35 days, in the second one—after 14 days that might be connected with slightly higher air temperature and longer day time. In the tank with LEDs, the growth of algae occurred until the 35th day in the first cycle and until the 28th day during the second cycle of measurement. The reason of this rapid change in particle diameter in the second measurement series might be occurrence of a few cycles of algae growth. The growth of algae culture consists of five main phases: lag phase, exponential phase, declining relative growth phase, stationary phase, and death phase (Price and Farag 2013; Dogaris et al. 2015). The occurrence of more than one cycle of algae growth and death might be connected with slightly higher air temperatures than occurred in the first measurement series. Optimal temperature for growth of most of the algae species is between 16 and 27 °C. Temperature below 16 °C may reduce the speed of the algae growth (Blinová et al. 2015). The slightly lower temperatures during the first experimental series could cause occurrence of one cycle of algae growth during 56 days of experiment.

Additional factor which has a huge impact on the algae aggregates formation is low concentration of nutrients in the solution. Single algae cells that occur in the wastewater in the case of lack of an optimal amount of nitrogen and phosphorus in an easily available form produce extracellular polymeric substances (EPS). EPS covers the cells surface and initiates adhesion of subsequent algae cells. This process leads to aggregates formation (Cook et al. 2007; McMinn and Lee 2018).

The formation of algae suspension in controlled conditions can be simulated by the Avrami’s theory. It is especially suitable to model the growth of algae in the initial and stable phase of growth. The modified Avrami equation can be used to describe dynamics of algae colonies growth by comparing this process to the mechanisms of crystals formation (Kuśnierz and Łomotowski 2015). As reported by Tran et al. (2013), this model can be also applied to describe the process of mortar surface colonization by photosynthetic organisms. The crystallization process is based on the precipitation of individual particles of substances dissolved in the solution around the crystallizing nuclei. In the case of algae, its single cell forms the nucleus of crystallization. Other single cells of algae that float freely in the depth of wastewater are being attached to the nucleus creating aggregates.

Determination of the fractal dimension of algae colonies may pay a key role in the systems using natural processes for the wastewater treatment. As presented by Burszta-Adamiak et al. (2009) and Valle et al. (2017), the particles with fractal dimension close to 1 have linear shape, with an increase to 2, they have a more developed surface, when particles with the Df around 3 are more concentrated and have more extensive surface (Valle et al. 2017). The fractal dimension may strongly affect the possibility of suspended solids removal from the sewage. Recognition of particle characteristics allow to assume the possibility of aggregates breakage and thus to choose efficient processes of particle removal (Jarvis et al. 2005).

## Conclusions

The study shows that the additional lighting of wastewater with use of the red and blue LEDs during the nighttime has a significant impact on algae growth.

In the tank with additional light source, the growth of algae colonies was faster than in the wastewater from the tank without artificial light especially during the first measurement series when the air temperature oscillated within 15–20 °C.

During the second measurement series, when the air temperature was slightly higher, the speed of algae growth was similar in both—wastewater with and without additional light source—and took 14 days for the first, most intensive growth phase.

The fractal dimension of algae aggregates in the wastewater illuminated with combination of red and blue LEDs reached slightly lower maximum values than in the wastewater without LEDs. In each measurement series, the fractal dimension of particles in the tank with lighting in the end of the experiment was about 33–43% lower than in the tank without LEDs. That indicates that in the wastewater without additional light source, the particles are more compacted while in the wastewater supported with artificial light, the particles are more linear.

The results obtained during the study confirms that using modified Avrami equation to predict algae growth is a good solution especially when the information about character of particles has to be provided in no time. It can have particular application in the selection of methods for removing algae from the treated wastewater.
